# Supplementary material for: Trapping IgE in a closed conformation by mimicking CD23 binding prevents and disrupts FcεRI interaction
Source: Nat Commun. 2018 Jan 2;9:7. doi: 10.1038/s41467-017-02312-7 (PMC5750235; doi:10.1038/s41467-017-02312-7)
Supplement: Supplementary file 1 — Supplementary information [file 41467_2017_2312_MOESM1_ESM.pdf]

Supplementary Figures:

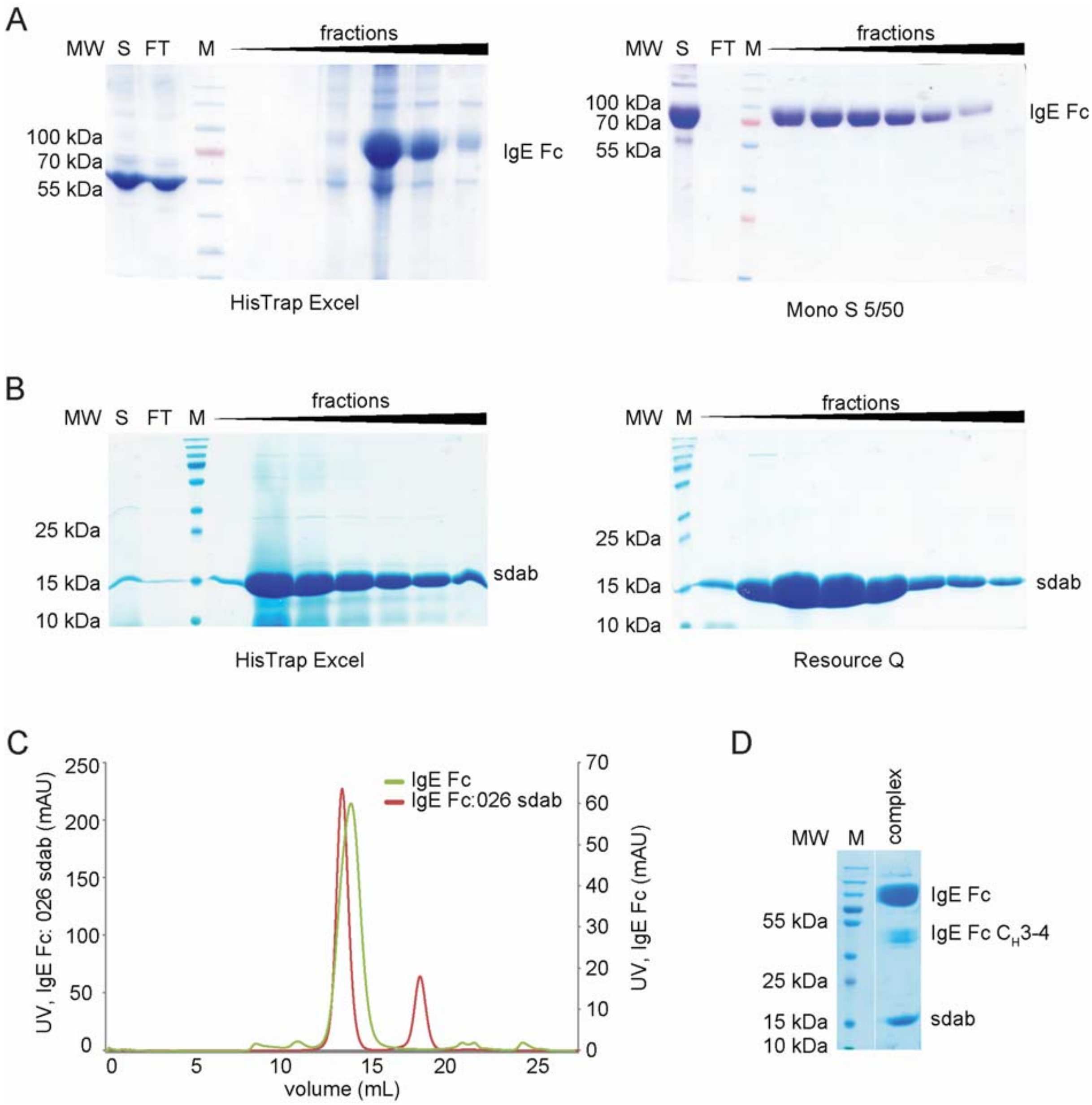

Supplementary Figure 1: Purification of the IgE Fc:026 sdab complex for crystallization.
A-B: Non-reducing SDS-PAGE analysis of the purification of the recombinant IgE Fc and the purification of the recombinant 026 sdab. C: Analytical size-exclusion chromatography of the IgE Fc and the complex with 026 sdab shows the complex formation. For the latter, the sdab and IgE Fc were mixed in a 4:1 molar ratio. D: Non-reducing SDS-PAGE analysis of the complex documents presence of the IgE Fc and the sdab. In addition a minor band at approx. 45 kDa demonstrates the presence of small amounts of the IgE Fc Cε3-4 fragement. S: supernatant, FT: flow through, M: marker, MW: molecular weight.

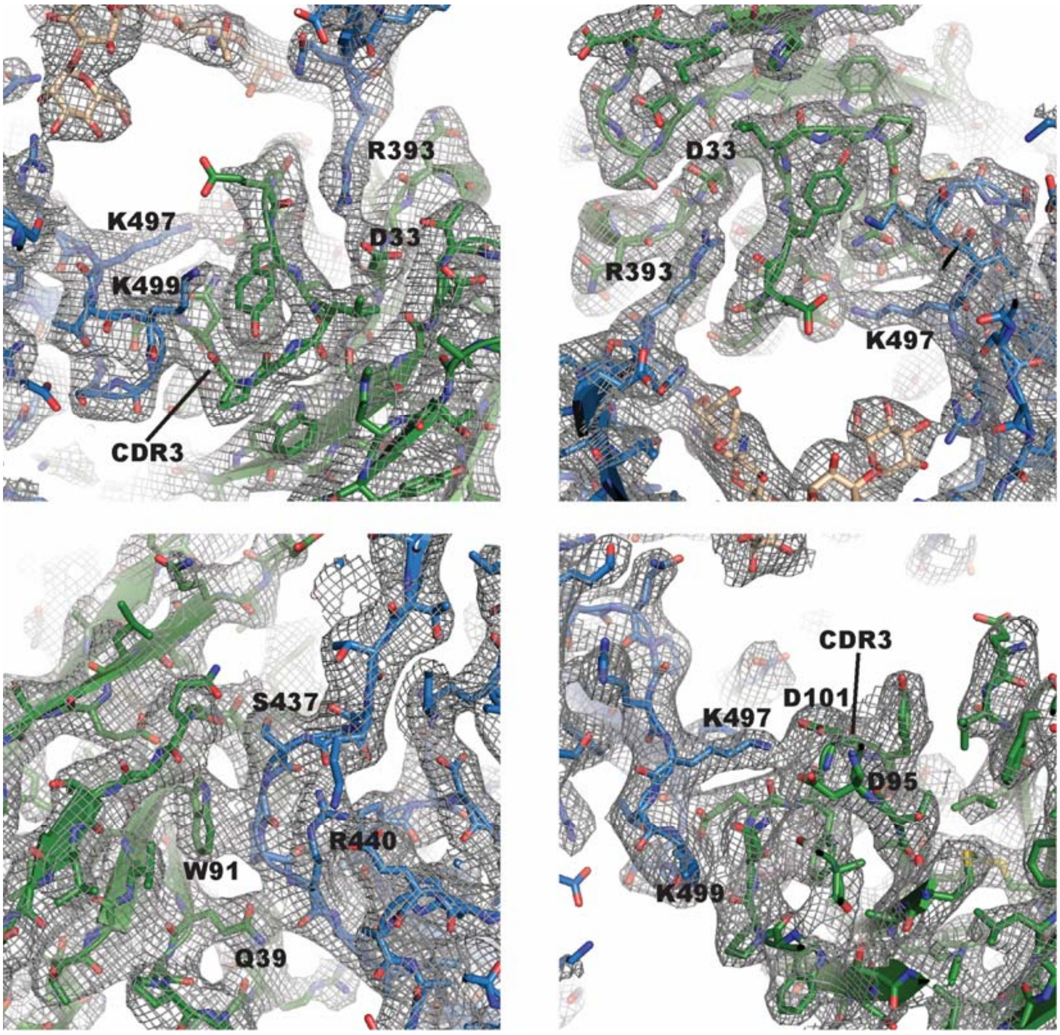

Supplementary Figure 2: Electron density from the intermolecular interface. A 2mF$_o$-DF$_c$ electron density map contoured at 1σ at different interface regions in the complex. The 026 sdab is shown in green and IgE Fc in blue.

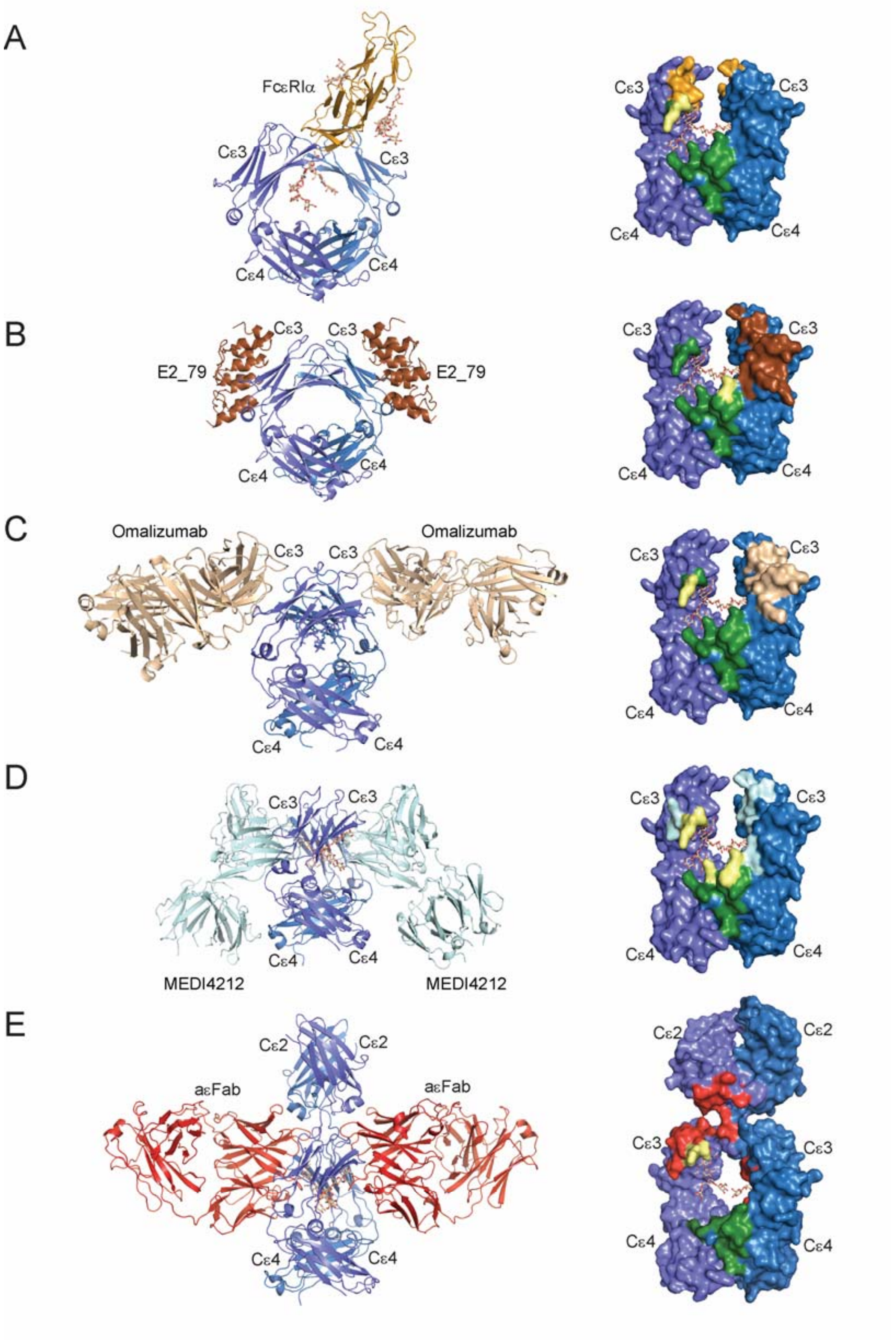

Supplementary Figure 3: The epitopes of several IgE binding molecules and their overlap with the 026 sdab epitope.

Cartoon representations are shown to the left with the IgE Fc in blue and other Fc binding proteins in other colours. To the right are displayed surface representations with the sdab epitope in green, the epitope of the other IgE binder in individual colours, and overlapping epitope residues in yellow. Comparison of the IgE Fc:026 sdab complex with the A: FcεRI complex (PBD ID: 1F6A), B: IgE Fc:E2_79 DARPin complex (PDB ID: 4GRG), C: IgE Fc:Omalizumab Fab complex (PDB ID: 5HYS), D: IgE Fc:MEDI4212 Fab complex (PDB ID: 5ANM), and E: another IgE Fc:anti-IgE Fab complex (PDB ID: 4J4P).

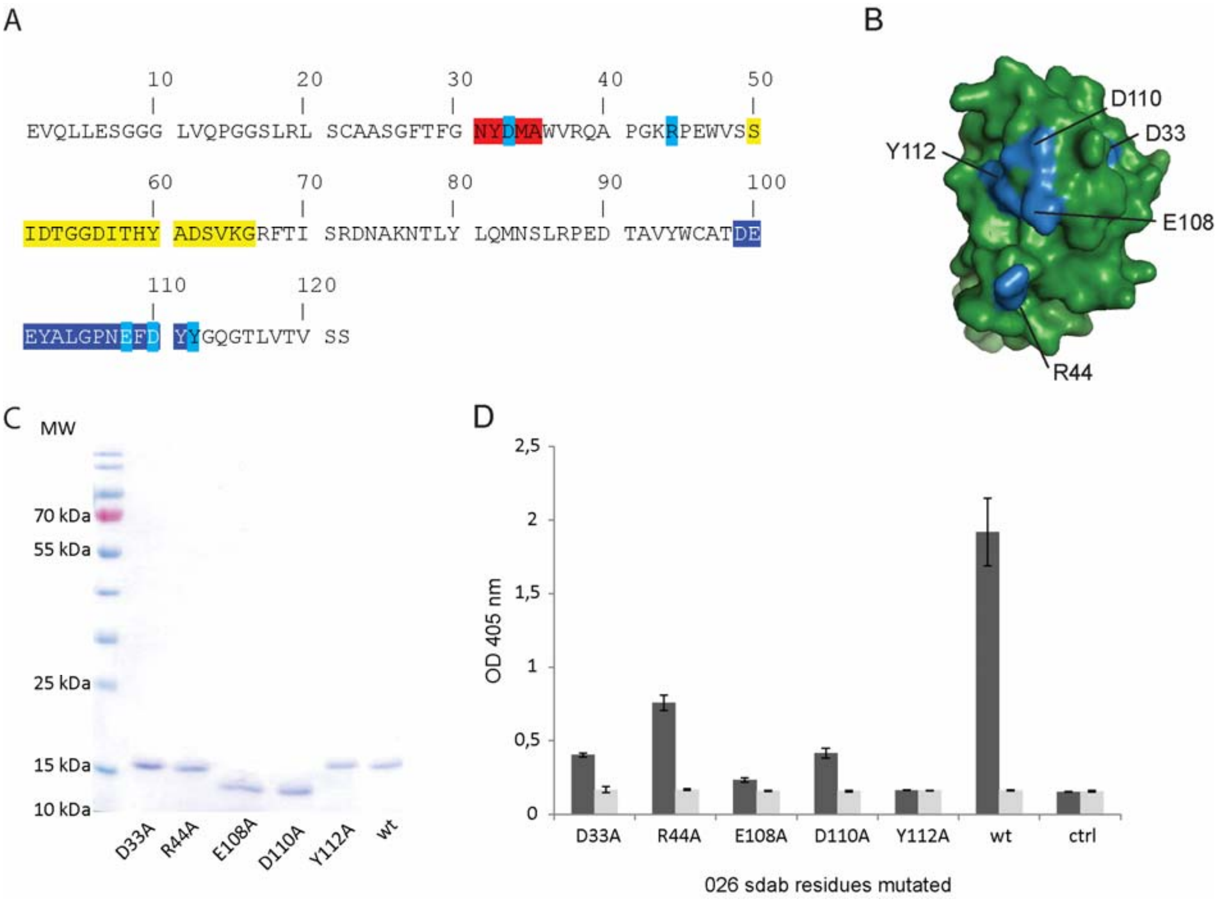

Supplementary Figure 4: Functional analysis of 026 sdab residues.
A: Consecutive sequence numbering of the 026 sdab showing the CDR1 in red, CDR2 in yellow and CDR3 in blue. Mutations are indicated in lightblue. B: Structure of the 026 sdab. Indicated are the mutations introduced for functional analysis. All mutations are present on the interface of the sdab and IgE Fc. C: SDS-PAGE analysis of sdab wild type and five mutants after purification. D: Immunoreactivity of the six sdab variants to IgE Fc was assessed by ELISA. Detection of His-tagged sdabs bound to immobilized IgE Fc was performed using an anti-his antibody conjugated to alkaline phosphatase. Data of a representative experiment are mean values and standard deviation of triplicates.

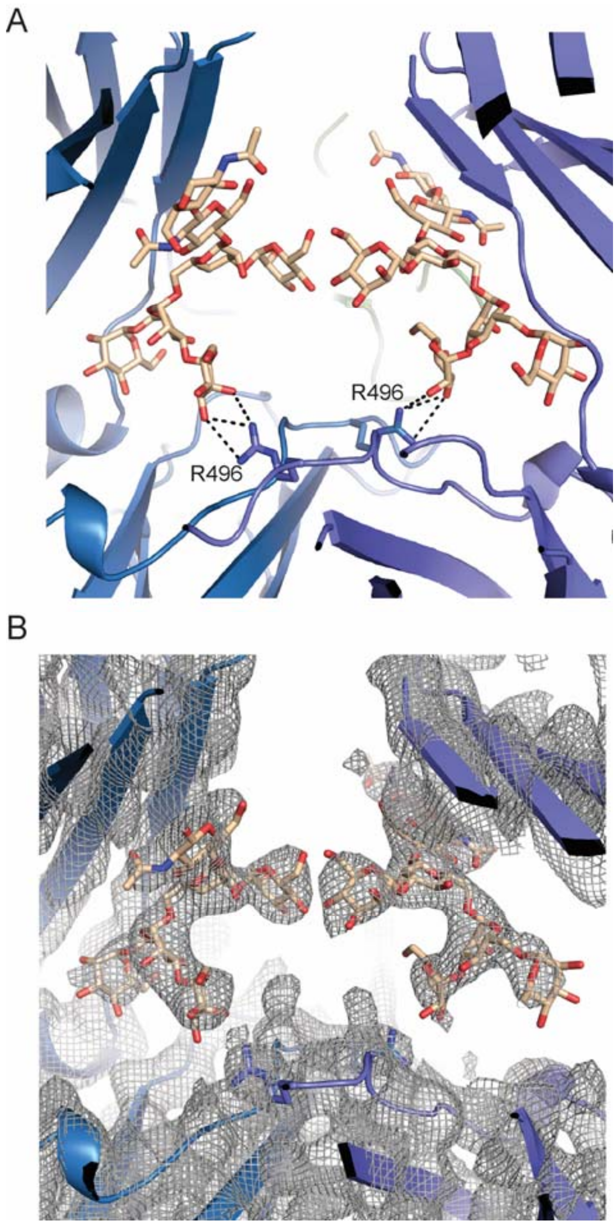

Supplementary Figure 5: The Ig Fc glycan in the complex.
A: Structure of the heptasaccharide at Asn394 of the IgE with putative hydrogen bonds between Arg496 and a mannose residue shown as black dotted lines. B: A $2mF_o$-DF_c omit map contoured at 1σ. The heptasacchride at Asn394 was omitted during calculations.

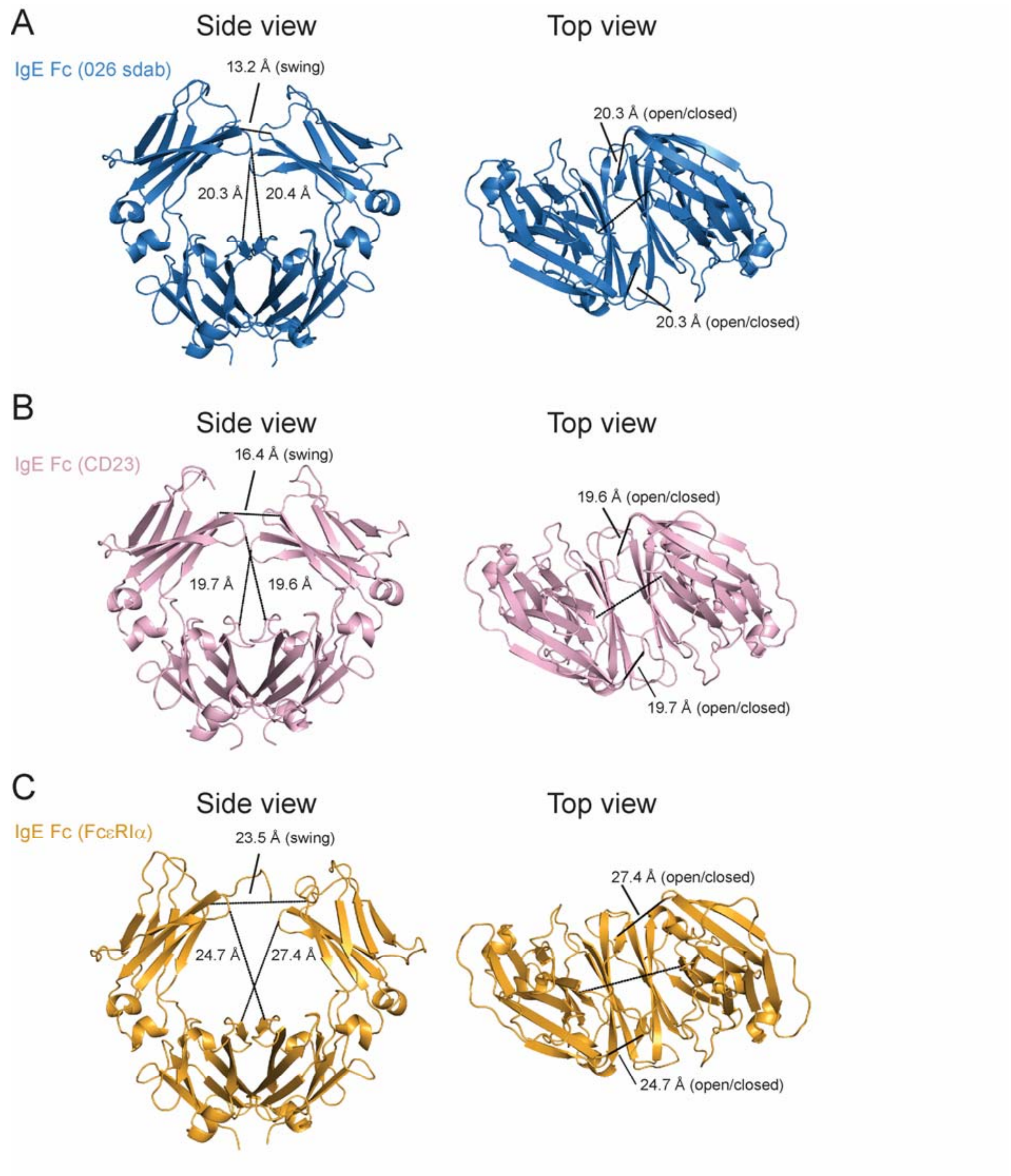

Supplementary Figure 6: The 026 sdab is bound to IgE Fc in closed conformation. Structures of IgE Fc in complex with 026, CD23 and FcεRI with distances (black lines) defining their "swing" and "open/closed" properties. The swing is quantitated as the distance between Cα atoms of residues 336 in the Fc dimer. Open/closed is defined by the distance between Cα atom of residue 394 and the Cα of residue 497 in the opposing chain [1]. A: The IgE Fc in complex with 026 sdab shown in top and side views. B-C: As in panel A, but displaying the IgE Fc:CD23 (panel B) and IgE Fc:FcεRI complex (panel C).

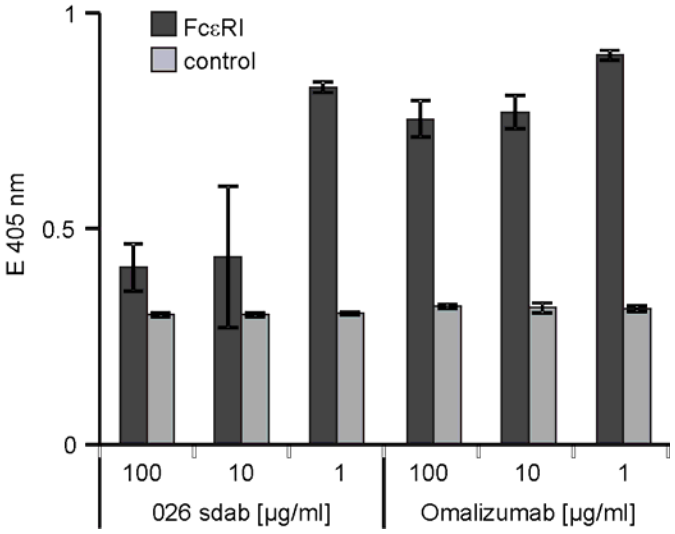

Supplementary Figure 7: Displacement of IgE from FcεRI.
Displacement of IgE from soluble FcεRI extracellular domains was assessed in ELISA. Immobilized FcεRI was incubated with IgE followed by incubation with 026 sdab and omalizumab and detection of remaining IgE as compared to a control without the antibody. Data are mean values and standard deviation of triplicates.

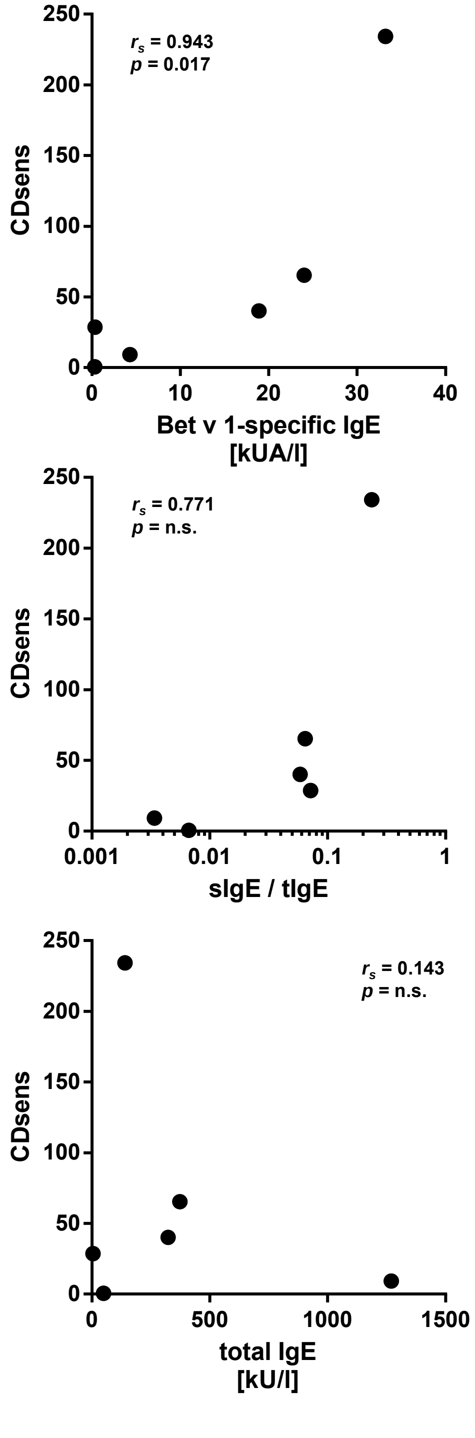

Supplementary Figure 8: Correlation of CDsens and IgE level.
CDsens was correlated with allergen-specific IgE (sIgE), the ratio of sIgE and and total IgE (tIgE), and tIgE serum concentrations alone for six patients with birch pollen allergy. Correlation analyses were performed by using the two-tailed Spearman's rank correlation coefficient. Differences were considered statistically significant at p values <0.05.

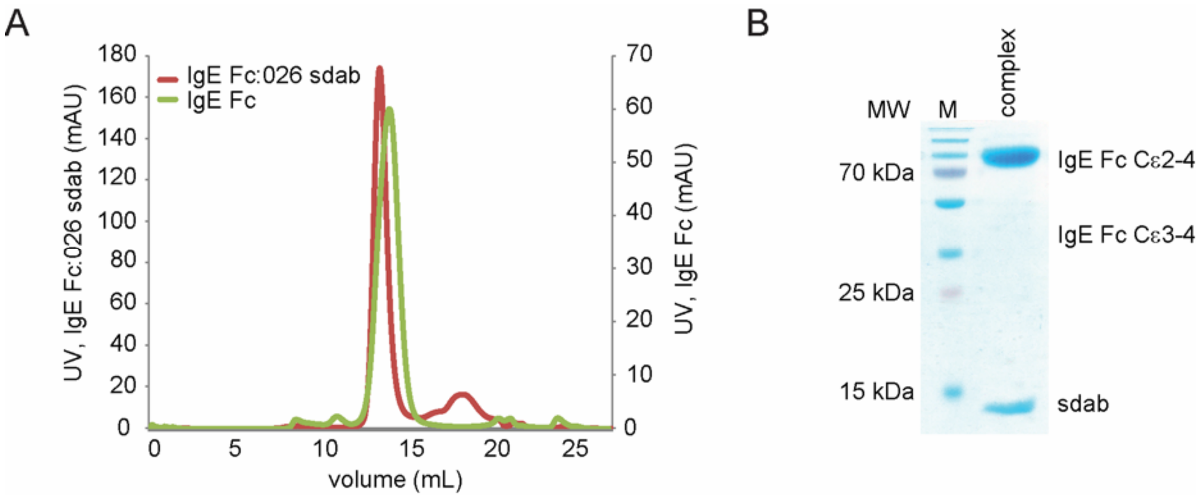

Supplementary Figure 9: Purification and complex formation for SAXS.
A: Analytical size-exclusion chromatography of the IgE Fc alone and in complex with the 026 sdab demonstrating formation of the complex. For the latter, 026 sdab and IgE Fc were mixed in a 4:1 molar ratio. B: Non-reducing SDS-PAGE analysis of the complex documents the presence of the IgE Fc and the sdab and removal of the truncated IgE Fc Cε3-4 by hydrophobic interaction chromatography.

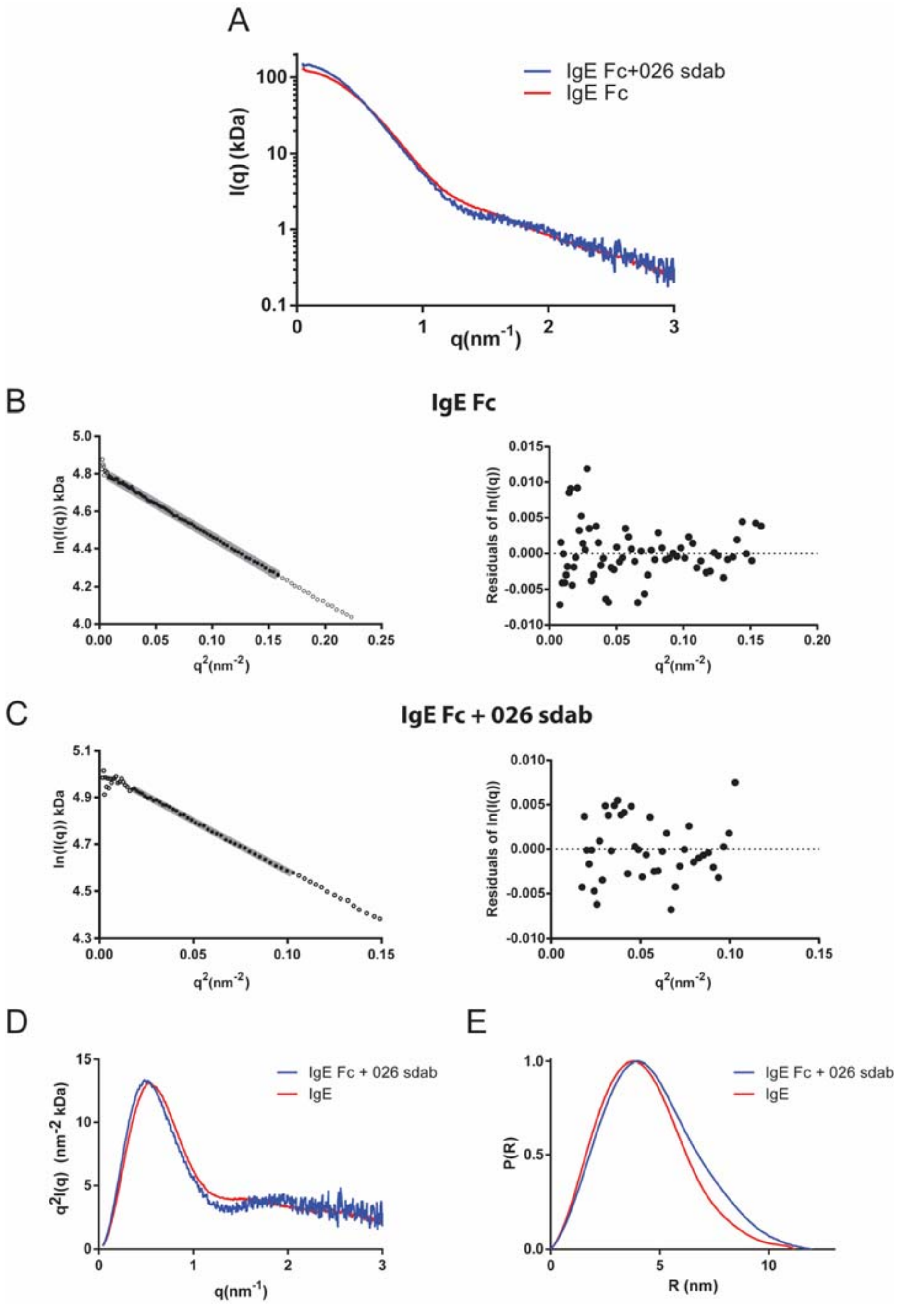

Supplementary Figure 10: Solution scattering analysis of IgE Fc and its complex with 026 sdab.

A: The scattering curves of IgE Fc Cε2-Cε4 alone (blue) and IgE Fc in complex with 026 sdab (red). B: The Guiner plot for IgE Fc (black), the linear fit (gray) and the residual plot of the linear fit. No sign of interparticle effects are present and the plot suggests a radius of gyration of 3.3 nm similar to values previously observed for IgE Fc [2]. C: As in panel B for the IgE Fc in complex with 026 sdab. The estimated radius of gyration is 3.6 nm. D: The Kratky plot of IgE Fc (red) and IgE Fc in complex with 026 sdab (blue). Both IgE Fc alone and in complex with 026 sdab are well folded and have limited flexibility. E: The pair-distance distribution function of IgE Fc (red) and IgE Fc in complex with 026 sdab (blue). A similar mass distribution is observed but with the complex being slightly larger than IgE Fc on its own. The D_{max} is estimated at 11 nm and 11.5 nm for IgE Fc and the complex, respectively.

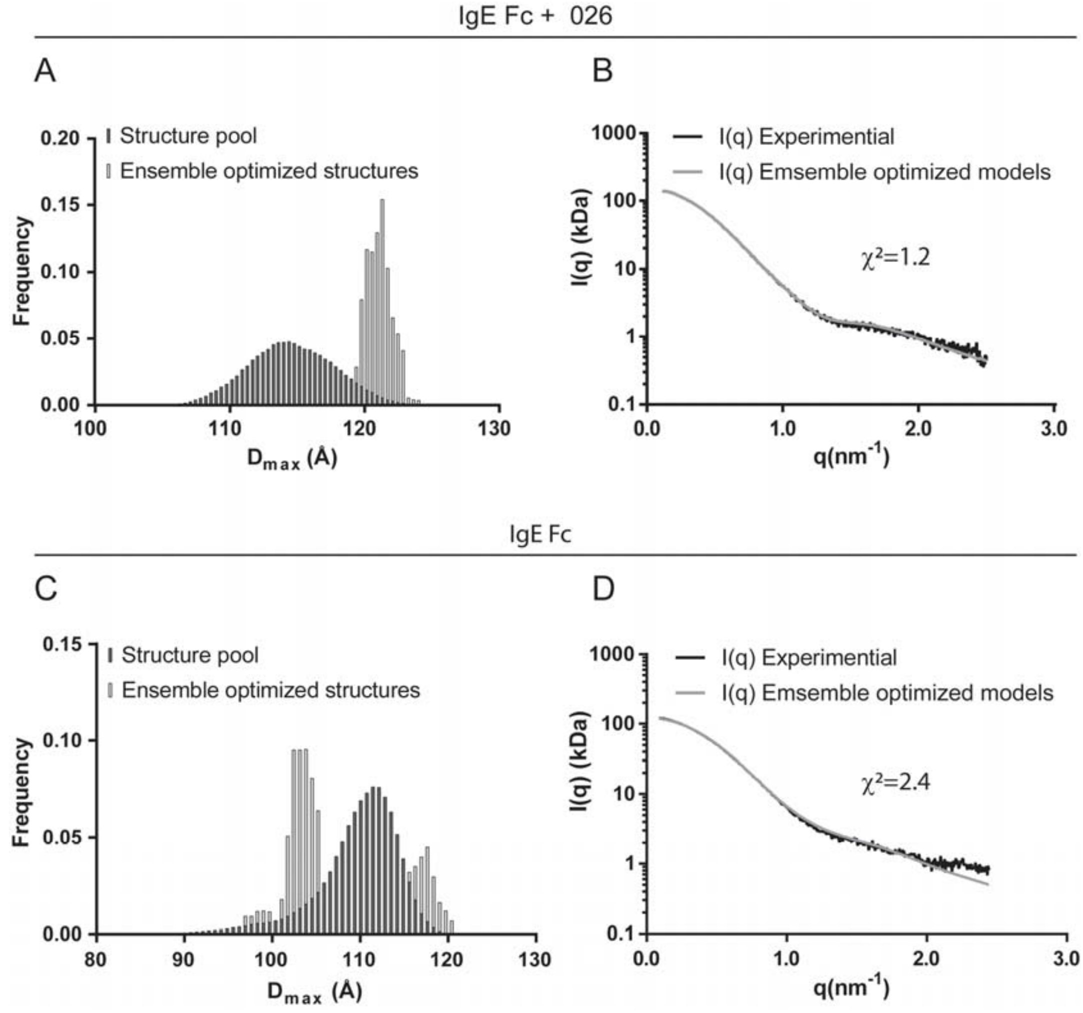

Supplementary Figure 11: EOM analysis of the IgE Fc:026 sdab complex and unbound IgE Fc.

A: Histograms of D_{max} values for the IgE Fc:026 sdab complex for the randomly generated pool of structures (dark grey) and the ensemble optimized structures (light grey) selected by EOM. B: Comparison of the scattering curve calculated from the ensemble optimized structures as output from GAJOE (light grey) to the experimential SAXS curve (black) for the IgE Fc:026 sdab complex. C-D: As panels A-B but for unbound IgE Fc.

Supplementary Tables:

Supplementary Table 1: Serological patient data

ID	allergen	tIgE [kU/L]	sIgE [kUA/L]
1	birch	1270	4.31
2	wasp venom	474	5.34
3	honeybee venom	44.4	6.34
4	birch	373	24.0
5	birch	323	18.9
6	birch	140	33.2
7	birch	49.7	0.33
8	birch	4.88	0.35

Supplementary Table 2: Data collection and scattering derived parameters

Data-collection parameters	IgE Fc:026 sdab	IgE Fc
Instrument	ESRF BM29	ESRF BM29
Wavelength (Å)	0.992	0.992
q range ($Å^{-1}$)	0.0033 - 0.50	0.0033 - 0.50
Exposure time pr. frame (s)	2	2
No. of frames averaged	10	10
Temperature (K)	277.15	277.15
Concentration range (mg ml^{-1})	1.7	2.2-9.0
Structural parameters†		
$I(0)$ [from $P(r)$]	123.1	92.3
R_g (Å) [from $P(r)$]	36.5	34.2
$I(0)$ (from Guinier)	123.3 +/- 0.11	92.3 +/- 0.078
R_g (Å) (from Guinier)	36.5 +/- 0.3	33.9 +/- 0.2
D_{max} (Å)	125	120
Molecular-mass determination†		
Molecular mass M_r [from $I(0)$] ‡	134	101
Calculated M_r from sequence	102	73
Software employed		
Data processing	*PyFAI/EDNA*	*PyFAI/EDNA*
Rigid-body modelling	CORAL	CORAL
EOM	Ranch & GAJOE	Ranch & GAJOE

†Reported for the 2.2 mg ml^{-1} measurement for IgE Fc. ‡Using BSA with I(0) = 65.98 and Mw = 72 kDa as a standard. Notice that the calculated molecular mass is based solely on the protein sequence and does not include the three glycans on each Fc ε-chain (Fig. 1A).

Supplementary Table 3: Sequences of the synthetic gene and mutational primer

Name	Sequence (5’-3’)
026 sdab (synthetic gene)	ccatggaggttcagctgctggaaagcggtggtggtctggttcagcctggtggtagcctgcgtctgagctgtgcagcaag cggttttacctttggtaattatgatatggcatgggttcgtcaggcaccgggtaaacgtccggaatgggttagcagcattgat accggtggtgatatcacacattatgccgatagcgttaaaggtcgttttaccattagccgtgataatgccaaaaataccctgt acctgcagatgaatagtctgcgtccggaagataccgcagtttattggtgtgcaaccgatgaagaatatgcactgggtccg aatgagtttgattattatggtcagggcaccctggttaccgttagctcagcggccgcagatc
026D33Afwd	tttggtaattatgctatggcatgggtt
026D33Arev	aacccatgccatagcataattaccaaa
026R44Afwd	gcaccgggtaaagctccggaatgggtt
026R44Arev	aacccattccggagctttacccggtgc
026E108Afwd	ctgggtccgaatgcgtttgattattat
026E108Arev	ataataatcaaacgcattcggacccag
026D110Afwd	ccgaatgagtttgcttattatggtcag
026D110Arev	ctgaccataataagcaaactcattcgg
026Y112Afwd	gagtttgattatgctggtcagggcacc
026T112Arev	ggtgccctgaccagcataatcaaactc

Supplementary References

1 Wurzburg, B. A. & Jardetzky, T. S. Conformational flexibility in immunoglobulin E-Fc 3-4 revealed in multiple crystal forms. *J Mol Biol* 393, 176-190, doi:10.1016/j.jmb.2009.08.012 (2009).

2 Beavil, A. J., Young, R. J., Sutton, B. J. & Perkins, S. J. Bent domain structure of recombinant human IgE-Fc in solution by X-ray and neutron scattering in conjunction with an automated curve fitting procedure. *Biochemistry* 34, 14449-14461 (1995).
